# A Suspected Case of Adhesion to the Iliac Vein During the Left Femoral Arthroplasty

**DOI:** 10.7759/cureus.60589

**Published:** 2024-05-19

**Authors:** Tomohiro Nakajima, Tsuyoshi Shibata, Yutaka Iba, Ima Kosukegawa, Nobuyoshi Kawaharada

**Affiliations:** 1 Cardiovascular Surgery, Sapporo Medical University, Sapporo, JPN; 2 Orthopedic Surgery, Sapporo Medical University, Sapporo, JPN

**Keywords:** shock, hipbone, iliac artery, iliac vein, femoral arthroplasty

## Abstract

An 85-year-old man underwent hemiarthroplasty for a left intertrochanteric femoral fracture at another hospital two years prior. While under outpatient monitoring, the left femur displacement occurred. Therefore, total hip arthroplasty of the left hip was scheduled. However, during acetabular cup insertion damage to the inner plate led to a sudden decrease in blood pressure from 120 to 60 mmHg. The physicians suspected a pelvic vascular injury and promptly stopped the procedure. In case of adhesion between the acetabular cup and the left iliac vein, intraoperative vascular damage would be repaired via endovascular intervention. Subsequently, orthopedic surgery was cautiously performed, taking into account the potential of a vascular injury. The surgery proceeded as planned without vascular intervention. This case involved a patient with suspected injury to the iliac vein and artery during acetabular cup placement. Following comprehensive enhanced CT and angiography tests, orthopedic surgery was performed in preparation for potential vascular damage, demonstrating the multidisciplinary approach to managing such cases.

## Introduction

Femoral intertrochanteric fractures in elderly patients often require surgical intervention. Treatment options include osteosynthesis and artificial femoral head replacement [1]. While minimally displaced femoral neck fractures may achieve favorable outcomes with less invasive osteosynthesis, significantly displaced fractures tend to require reoperation due to complications in approximately 20-30% of cases [2].

In hip arthroplasty, an acetabular cup must be implanted in the acetabular cavity. The anatomical location of the cavity near the trunk poses a risk to the surrounding structures, particularly the internal and external iliac arteries and veins.

## Case presentation

**Figure 1:**
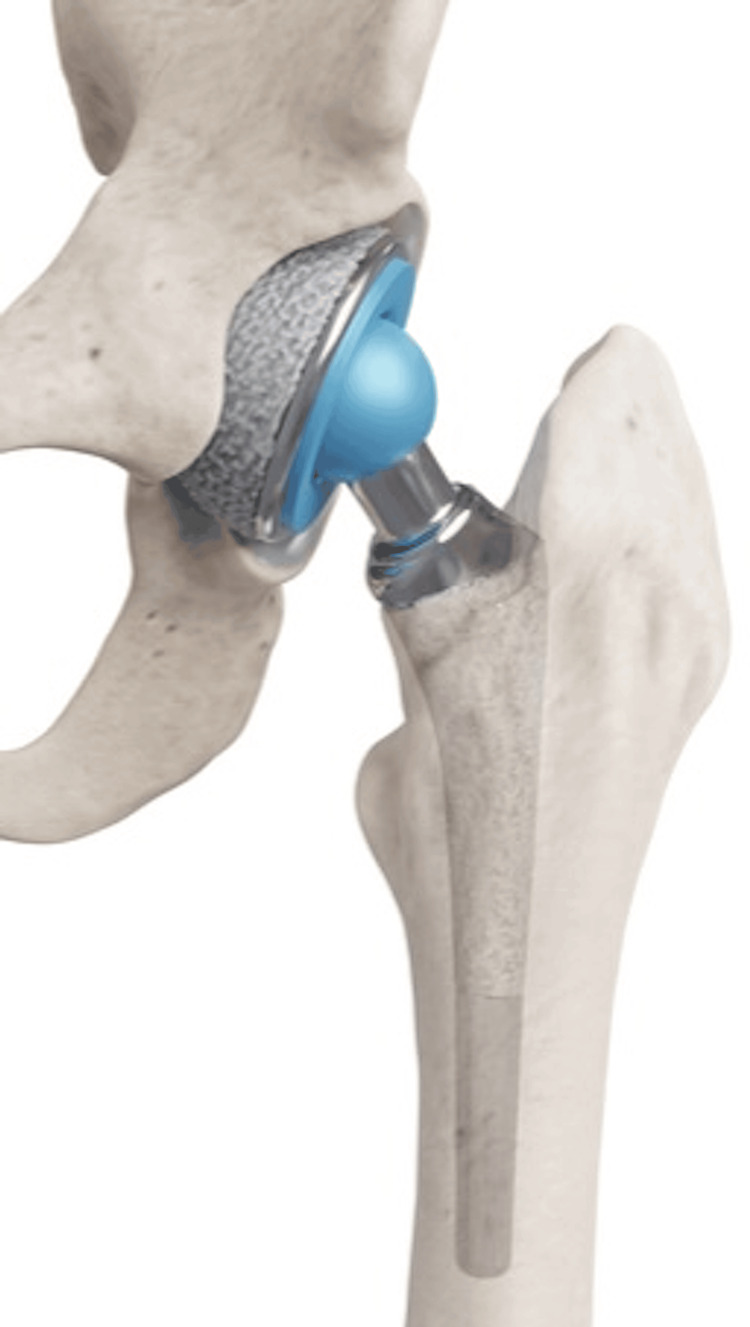
Schema of procedure A standard procedure schema for hip arthroplasty involved placing the cup in the acetabular rim

An 85-year-old man underwent open reduction and internal fixation (ORIF) surgery for a left femoral trochanteric fracture at another hospital two years prior. During the follow-up, progressive misalignment of the left femoral head was observed. The orthopedic surgeon determined that correction was not feasible with another ORIF; hence, a total hip arthroplasty was scheduled. A standard total hip arthroplasty schema is shown in Figure 1.

**Figure 2:**
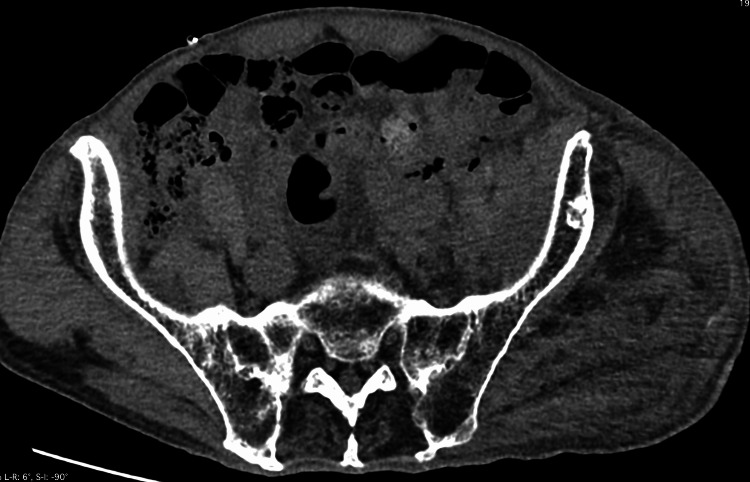
Computed tomography image The day after the first operation computed tomography revealed no intrapelvic bleeding

Under general anesthesia, the surgical procedure began with screw removal and resection of the femoral head on February 7th, 2024. However, the inner wall of the acetabulum was damaged during cup insertion into the acetabular rim. This was likely due to the patient’s brittle bones and the impact of the surgical technique. This resulted in a positional shift and a drop in blood pressure to 60 mmHg, prompting suspicion of vascular injury within the pelvis. Consequently, the procedure was halted, and the incision was closed to allow stabilization. The patient's hemoglobin level was 12.5 g/dL on preoperative blood draw and decreased to 6.7 g/dL due to intraoperative bleeding. The patient's hemoglobin level recovered to 10.5 g/dL by red blood cell transfusion. The day after this operation computed tomography revealed no intrapelvic bleeding (Figure 2).

**Figure 3:**
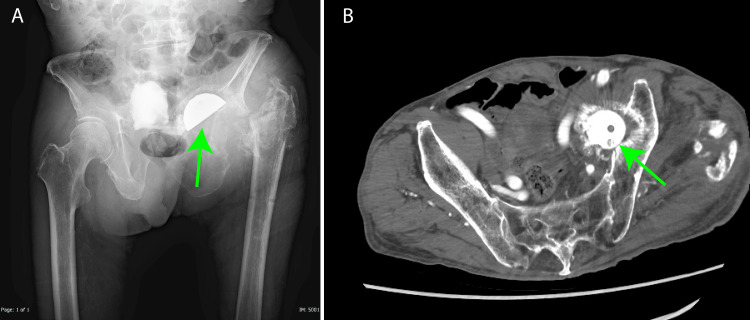
Preoperative images A) The joint cup is displaced medially beyond the acetabular cap (green arrow), as shown in the X-ray, and the left femoral bone head was already resected B) Enhanced computed tomography image revealed a cup located in the pelvis (green arrow)

After recovery with red blood cell transfusions, it was determined that reoperation at the previous facility would be difficult. It was considered better to transfer to a facility capable of performing vascular surgery. On March 15th, 2024, the patient was transferred to our hospital with vascular surgery capabilities. Thirty days post-surgery at the previous hospital, radiographs taken after the transfer revealed a displacement of the acetabular cup beyond the acetabular rim and into the pelvic cavity (Figure 3A). A plain CT tomography confirmed that the cup was within the pelvic cavity (Figure 3B).

**Figure 4:**
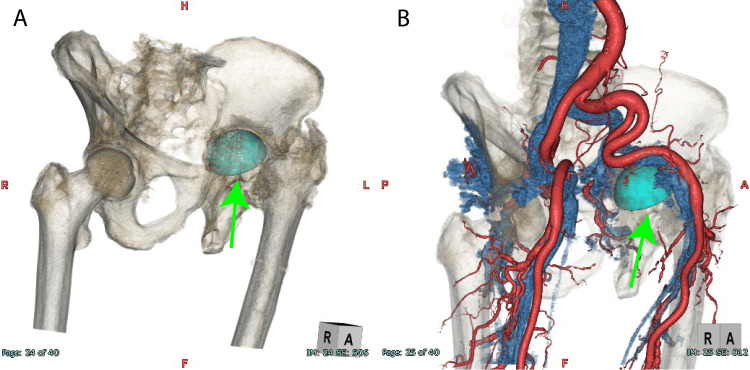
Computed tomography volume rendering images A) The cup is in the pelvis (green arrow) B) The left external iliac vein and cup are in close proximity (green arrow)

A contrast-enhanced CT revealed penetration of the acetabular rim, suggesting its proximity to the left external iliac vein (Figures 4A-4B).

**Figure 5:**
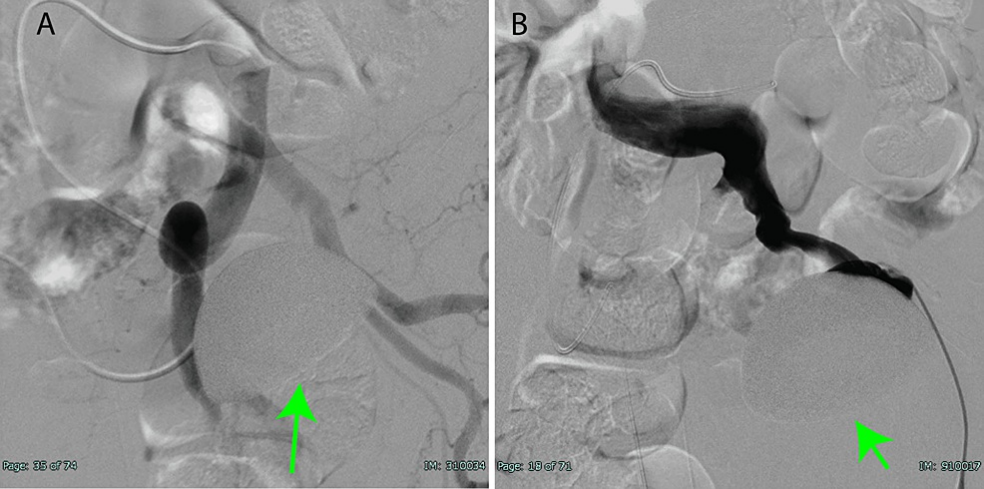
Angiography images from posterior to anterior A) Iliac arteriography showed no evidence of iliac artery compression by the cup (green arrow indicating the cup) B) Venography showed that the iliac vein was adjacent to the acetabular cup (green arrow indicating the cup)

A vascular angiogram examined the relationship between the acetabular cup and the pelvic blood vessels (Figures 5A-5B). Although there was no evidence of arterial compression, the cup compressed the left iliac vein, indicating a potential adhesion. The orthopedic and vascular surgery teams planned the surgical approach based on these results. The plan involved the orthopedic team proceeding with the scheduled hip arthroplasty, with immediate endovascular support in the event of vascular injury.

The surgery was performed under general anesthesia on March 31st, 2024. A 4Fr sheath was inserted into the left femoral artery and vein to prepare for the endovascular intervention. The patient was placed in the lateral decubitus position for total hip arthroplasty. Although some resistance was experienced when removing the previously placed acetabular cup, it was successfully extracted without significant changes in blood pressure or hemoglobin levels.

**Figure 6:**
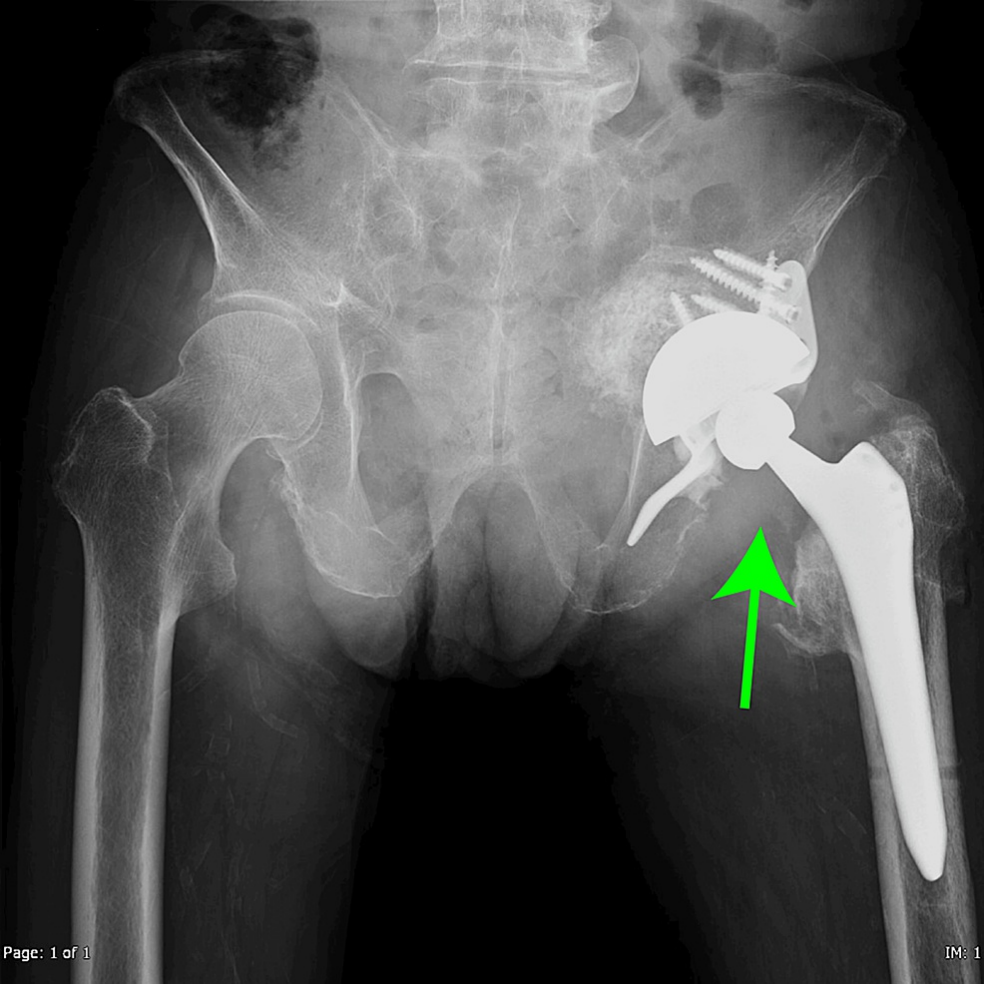
Postoperative image Artificial hip joints are implanted in the proper position (green arrow)

The acetabular rim was reconstructed, and total hip arthroplasty was planned. Postoperative radiography revealed no complications, and the patient was discharged on postoperative day 14 (Figure 6). This case presents a scenario where the proximity of the acetabular cup to the iliac veins and arteries posed a risk of vascular injury. After careful preoperative planning and the involvement of the vascular surgery team, the orthopedic procedure was completed without vascular complications. The patient was discharged on postoperative day 14 and could walk independently.

## Discussion

Femoral neck fractures are common among the elderly and, if not properly treated, can lead to prolonged immobility or even a permanent bedridden status [3]. Early mobilization and rehabilitation are crucial for treating femoral neck fractures in older adults, making surgical treatment the preferred choice over conservative therapy, which is often time-consuming [4]. Thus, reliable and quicker methods should be focused on to minimize the recovery period, even if they result in a slight reduction in ambulatory function.

Two primary surgical options are available for the treatment of femoral neck fractures: open reduction, internal fixation, and hemiarthroplasty with artificial femoral heads. The common complications of these surgeries include dislocation, infection, deep vein thrombosis, and nerve injury. However, reports on cup migration are rare [5].

In this case, based on the postoperative CT showing no hematoma within the pelvic cavity, we considered that the intraoperative bleeding and hypotension were likely due to extrapelvic hemorrhage from the pelvic fracture. Hemostasis was achieved by compression, stabilizing the patient's condition. However, the CT scan showed that the pelvic cup appeared to be protruding into the pelvic cavity and adhering to the iliac vein or iliac artery. A comprehensive series of imaging studies were conducted, including contrast-enhanced CT, venography, and arteriography, to evaluate the relationship between the cup and pelvic vessels. The initial assessment conducted at the previous hospital before the transfer suggested the possibility of pelvic vascular injury. However, given that a significant pelvic vascular injury would require immediate transfer, the progression observed in this case was deemed unlikely. Additionally, the absence of a vascular surgeon at the previous hospital made it difficult to confirm the vascular injury. Consequently, the orthopedic surgeon at the previous hospital suspected pelvic vascular injury, which led to the events described in this case.

To prepare for the surgery, protocols similar to those used for trauma-related pelvic fractures were followed [6]. The surgical team was prepared to insert a covered stent via endovascular intervention in case of venous or arterial damage and surgical instruments for direct vascular repair were prepared for the patient in the case of uncontrollable bleeding. Fortunately, no significant bleeding or vascular injury occurred during the extraction of the migrated cup, allowing orthopedic surgery to be completed without further vascular intervention.

An alternative to our protocol for mitigating the risk of vascular injury during hip hemiarthroplasty could have involved preemptively inserting an endovascular stent before proceeding with the hip replacement. While this approach has the advantage of preventing bleeding due to vascular injury, it also has the disadvantage of potentially placing an unnecessary stent in cases of no adhesion or risk to the blood vessels during the procedure.

Therefore, in this case, after thorough discussions between the vascular and orthopedic teams, we chose to follow our protocol. It is worth noting that the protocol choice may vary depending on the case or the available resources at a given facility, highlighting the need for tailored approaches.

## Conclusions

We encountered a case where the acetabular cup inserted into the acetabulum protruded into the pelvic cavity more than expected during a femoral neck fracture treatment. This raised concerns regarding potential injury to the iliac artery or vein, prompting detailed preoperative imaging to evaluate the spatial relationship between the cup and the blood vessels.

We meticulously prepared for endovascular intervention to address possible vascular injury before proceeding with orthopedic surgery, which involved hip hemiarthroplasty. Fortunately, no vascular injury occurred during the procedure, which allowed the surgery to be successfully completed.
